# MiR-206 suppresses epithelial mesenchymal transition by targeting TGF-β signaling in estrogen receptor positive breast cancer cells

**DOI:** 10.18632/oncotarget.8233

**Published:** 2016-03-21

**Authors:** Kai Yin, Wenjin Yin, Yaohui Wang, Liheng Zhou, Yu Liu, Gong Yang, Jianhua Wang, Jinsong Lu

**Affiliations:** ^1^ Department of Breast Surgery, Fudan University Shanghai Cancer Center, Shanghai, China; ^2^ Department of Oncology, Shanghai Medical College, Fudan University, Shanghai, China; ^3^ Breast Cancer Center, Renji Hospital, School of Medicine, Shanghai Jiaotong University, Shanghai, China; ^4^ Cancer Institute, Fudan University Shanghai Cancer Center, Fudan University, Shanghai, China; ^5^ Central Laboratory, The Fifth People's Hospital of Shanghai, Fudan University, Shanghai, China

**Keywords:** breast cancer, miRNA, epithelial mesenchymal transition, TGF-β, migration

## Abstract

**Background:** Previous reports have shown a mutual negative feedback loop between microRNA (miR)-206 and estrogen receptor (ER) expression. Furthermore, decreased miR-206 expression in breast cancer (BC) is associated with the advanced clinical stage and lymph node metastasis. However, its role and the mechanism underlying the migration and invasion of ER positive BC remain unclear.

**Results:** In this study, miR-206 was stably transfected into ER positive cell lines MCF-7 and T47D to investigate the effect of miR-206. The results showed that miR-206 overexpression markedly impaired the migration and invasive abilities of these cells, followed by suppression of the epithelial mesenchymal transition (EMT). Mechanistic analyses showed that miR-206 inhibited the autocrine production of transforming growth factor (TGF)*-*β as well as the downstream expression of neuropilin-1 (NRP1) and SMAD2, responsible for the decreased migration, invasion, and EMT in these cells.

**Conclusions:** Our data demonstrate that miR-206 inhibits TGF-β transcription and autocrine production, as well as downstream target genes of EMT. Restoring miR-206 expression may provide an effective therapeutic strategy for ER positive BC.

## BACKGROUND

Breast cancer (BC) is the most common cancer among women and is still the second leading cause of cancer-related deaths among women in the United States, accounting for 29% of all new female cancer cases and 15% of all female cancer deaths [1]. Although the incidence rates vary from the lowest in Asia and Africa to the highest in North America, a dramatic increase of incidence has been observed in Asian countries over recent years, especially in China [2].

Estradiol is a key promoter for the carcinogenesis of BC, which enhances cell proliferation mainly through an estrogen receptor (ER) α dependent mechanism. ERα mediated estradiol signaling facilitates DNA mutations and the loss of wild type alleles of the tumor suppressor, thereby resulting in cancer development [3]. These mechanisms suggest a crucial role of ERα in BC carcinogenesis. However, the mechanism underlying the development of ER positive BC still remains unknown.

Previous studies have identified a large group of microRNAs (miRNAs) differentially expressed among cancer tissues compared to the normal tissues [4, 5]. Different subtypes [5–7], and highly metastatic BC cells compared to the wild type parental BC cells [8] were also reported. Among these miRNAs, miR-206 is an important ER associated miRNA, which is decreased in ER positive BC compared to the ER negative subtype. Adam et al. identified and validated miR-206 as an ERα negative regulator, and an estradiol activator could inhibit the expression of miR-206 [9]. Several studies also demonstrated that miR-206 was downregulated in ERα positive BC, indicating miR-206 expression is inversely correlated with ERα [10, 11]. The possible negative feedback loop between miR-206 and ERα implies an important role of miR-206 in ERα positive BC. Tavazoi et al. [8] used microarray analyses to define the inhibitory effects of miR-335 and miR-206 on the migration and invasion of highly metastatic BC cell lines. They showed a decreased median survival in patients whose primary tumors displayed low expression of miR-335 and miR-206, but their study focused on miR-335 and did not further determine the mechanism of miR-206 inhibition of metastasis [8]. Although the relationship between ERα and miR-206 has been reported, no studies have focused on their inhibitory effects for the metastasis of ER positive BC. This subset of BC comprises the majority of newly diagnosed breast cancer. In the current study, we determined the mechanism by which miR-206 regulates the migration and invasion of ER positive BC.

Three transforming growth factor (TGF)-β related signaling genes, *NRP1*, *SMAD2*, and *SMAD4*, are predicted as candidate miR-206 downstream targets using bioinformatics analyses. Our data indicated that miR-206 inhibited the expression of these genes, as well as the expression and autocrine activity of TGF-β. These effects could contribute to the migration and invasion of BC cells. Therefore, our results suggest that restoring miR-206 expression may impair the ability of migration and invasion of ER positive BC.

## RESULTS

### MiR-206 overexpression inhibits migration and invasion of ER positive BC cell lines

To explore the effects of miR-206 on migration and invasion of ER positive BC, we transfected MCF-7 and T47D cells with pre-miR-206 to overexpress miR-206 in these ER positive breast cancer cell lines (noted as MCF7-miR-206E and T47D-miR-206E). The overexpressions were confirmed by quantitative polymerase chain reaction (qPCR) analyses (Figure S1).

**Figure 1:**
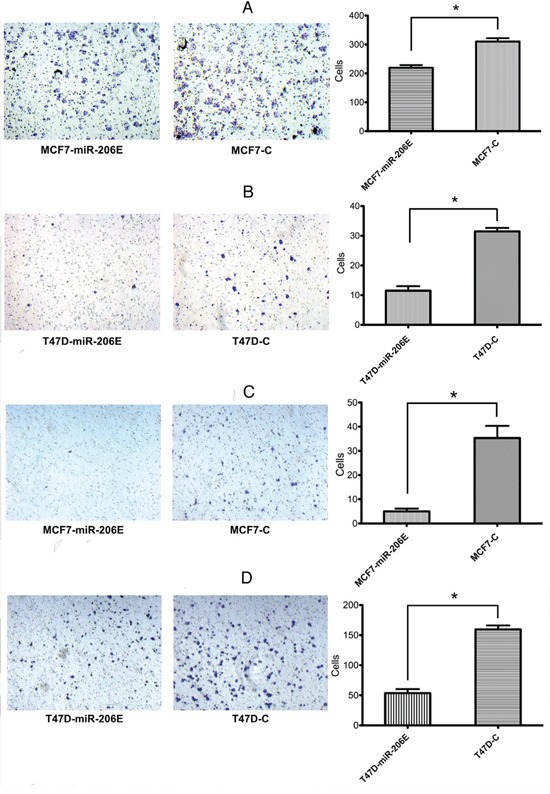
MicroRNA (miR)-206 suppresses the estrogen receptor (ER) positive breast cancer cell migration and invasion **A, B.** Transwell® migration assays showed that overexpression of miR-206 significantly suppressed cell migration in both MCF-7 and T47D cells (*P < 0.05). Experiments were performed in triplicate, and representative images of invaded cells are shown. Mean ± SD. **C, D.** Matrigel^®^ invasion assays showed that overexpression of miR-206 significantly suppressed cell invasion in both MCF-7 and T47D cells (*P < 0.05). Experiments were performed in triplicate, and representative images of invaded cells are shown. Results are expressed as mean ± SD.

The miR-206 effects on migration and invasion were assayed using Transwell^®^ chambers without or with Matrigel^®^, respectively. The results showed that the migration was significantly reduced in MCF7-miR-206E and T47D-miR-206E cells compared with the respective controls (Figure 1A and 1B). Similar effects were also observed in invasion assays (Figure 1C and 1D). Therefore, these findings suggest that miR-206 overexpression inhibits migration and invasion of ER positive BC cell lines.

### MiR-206 suppresses epithelial mesenchymal transition (EMT) through targeting TGF-β signaling

**Figure 2:**
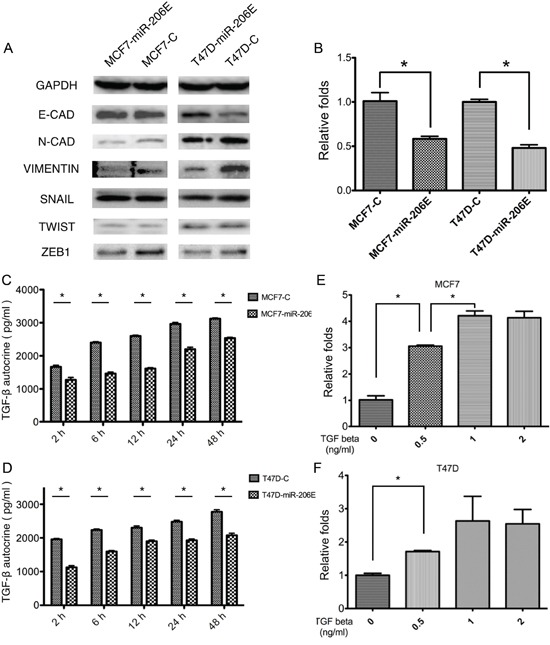
MicroRNA (miR)-206 suppresses the epithelial mesenchymal transition (EMT) by targeting transforming growth factor (TGF)-β transcription and autocrine expression **A.** Overexpression of miR-206 significantly suppressed the mesenchymal cell markers, N-cadherin and vimentin, as well as the EMT transcription factor ZEB1 in both MCF-7 and T47D cells. **B.** Overexpression of miR-206 significantly inhibited TGF-β transcription levels in both MCF-7 and T47D cells (*P < 0.05). Experiments were performed in triplicate. TGF-β mRNA expression was normalized to GAPDH and depicted as the mean ± SD. **C, D.** Overexpression of miR-206 significantly inhibited autocrine TGF-β expression in both MCF-7 and T47D cells (*P < 0.05). Experiments were performed in triplicate. Data are represented as mean ± SD. **E, F.** Both MCF-7 and T47D cells were treated with TGF-β1 at the indicated concentrations (0, 0.5, 1, 2 ng/mL), and miR-206 expression levels significantly increased with a TGF-β1 concentration-dependent manner when TGF-β1 concentration varied from 0–1 ng/mL in MCF-7 cells and varied from 0-0.5 ng/mL in T47D cells (*P < 0.05), and reached maximum levels with TGF-β1 concentrations of more than 1 ng/mL. Experiments were performed in triplicate. The miR-206 expression was normalized to U6 and depicted as the mean ± SD.

Western blot analyses were done to further investigate the mechanism of how miR-206 regulates the migration and invasion of ER positive BC (Figure 2A). The expression of E-cadherin protein, an epithelial cell marker, was increased in MCF7-miR-206E and T47D-miR-206E cells. The mesenchymal markers N-cadherin and vimentin were decreased in these cells, suggesting that miR-206 overexpression in the ER positive BC cell may be responsible for EMT, and that this could regulate migration and invasion. Moreover, the EMT transcription factor ZEB1 was inhibited in MCF7-miR-206E and T47D-miR-206E cells compared with their respective controls (Figure 2A).

**Figure 3:**
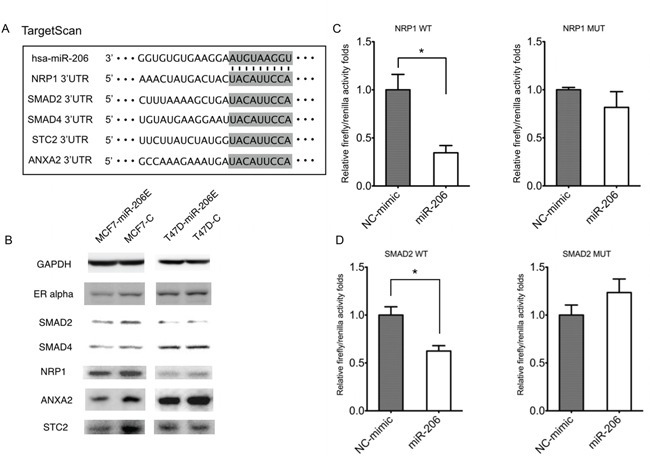
MiR-206 targets TGF-β signaling genes **A.** Prediction of miR-206 target genes using the TargetScan algorithm. **B.** The predicted miR-206 target gene expressions of *NRP1*, *SMAD2*, *ANXA2*, and *STC2* were downregulated by miR-206 overexpression. **C.** The 293T cells were co-transfected with wild type *NPP1* 3′-UTR luciferase construct (NRP1 WT), or the construct containing mutations in the predicted miR-206-binding site (NRP1 MT), and either miR-206 mimics or the NC-mimic. Luciferase expression was normalized to *Renilla* luciferase and depicted as the mean ± SD. **D.** The293T cells were co-transfected with wild type *SMAD2* 3′-UTR luciferase construct (SMAD2 WT), or the construct containing mutations in the predicted miR-206-binding site (SMAD2 MT), and either miR-206 mimics or the NC-mimic. Firefly luciferase expression was normalized to *Renilla* luciferase and depicted as the mean ± SD. The unpaired two-tailed Student's *t*-test was used in C, D) to determine the significance of differences between the two groups. Significance is presented as *P < 0.05.

Next, we determined which pathway was involved in the process of EMT. Interestingly, using the TargetScan algorithm, the three genes *NRP1*, *SMAD2*, and *SMAD4* of the TGF-β signaling factor pathway, were predicted target genes of miR-206 (Figure 3A). These results suggested that this pathway may play a role in miR-206 expression. Therefore, we determined how the expression of TGF-β was regulated by miR-206. The results showed that miR-206 overexpression in both MCF-7 and T47D cells suppressed TGF-β mRNA expression (Figure 2B). This finding was further confirmed at the protein level by an enzyme-linked immunosorbent assay (ELISA), using differing culture times (Figure 2C and 2D).

The effect of miR-206 expression upon exogenous TGF-β1 stimulation was also investigated. The results showed that miR-206 expression was significantly upregulated upon exogenous TGF-β1 stimulation (Figure 2E and 2F). Taken together, these results suggest that inhibition of TGF-β signaling by miR-206 overexpression results in the suppression of the EMT in ER positive BC cells. In contrast, exogenous TGF-β1 stimulation promotes miR-206 expression, which can inhibit the autocrine expression of TGF-β, suggesting that negative feedback regulation of TGF-β may be mediated by miR-206.

### MiR-206 inhibits *NRP1* and *SMAD2* gene expression by directly binding to their 3′-UTRs

As shown in Figure 3A, the expression of key genes belonging to the TGF-β signaling pathway family, including *NRP1*, *SMAD2*, and *SMAD4* may be reduced by miR-206. We further investigated the mechanism of how miR-206 regulates these genes. The results showed that the expression of the *NRP1*, *SMAD2*, *STC2*, and *ANXA2* genes were inhibited by miR-206 overexpression in MCF-7 and T47D cells (Figure 3B). To determine if *NRP1* and/or *SMAD2* are the direct target genes of miR-206, the wild type or mutant 3′-URT sequences of these genes were cloned downstream of the firefly luciferase coding region in the GV272 basic reporter vector, and were then co-transfected with the miR-206 mimic or negative control (NC)-mimic. The luciferase activities of the *NRP1* and *SMAD2* wild type 3′-UTR expression vectors were significantly reduced by miR-206 and rescued by their mutant 3′-UTR vectors (Figure 3C and 3D). This result suggests that *NRP1* and *SMAD2* are downregulated by miR-206 through directly binding to their 3′-UTRs.

### The inhibitory effects of miR-206 on migration and invasion are reversed by exogenous TGF-β stimulation

**Figure 4:**
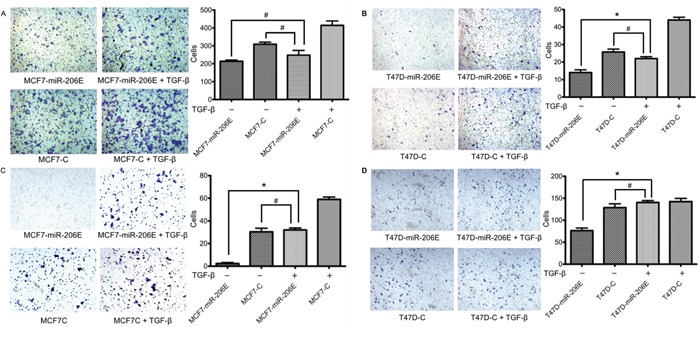
Exogenous TGF-β1 stimulation of miR-206 overexpressing cells restores their migration and invasion capabilities MiR-206 expressing cells were treated with 20 ng/mL TGF-β1 for 3 days. **A, B.** Migration assays showed that cell migration in both MCF7-miR-206E and T47D-miR-206E cells was restored when compared to the non-treated negative control cells. **C, D.** Matrigel® invasion assays showed that cell invasion in both MCF7-miR-206E and T47D-miR-206E cells was restored when compared to the non-treated negative control cells. Significance is presented as *P < 0.05, and ^#^*P* > 0.05. Experiments were performed in triplicate, and representative images of invaded cells are shown. Data are depicted as the mean ± SD.

Based on the above findings, we investigated whether the inhibitory effects of miR-206 on migration and invasion could be restored by TGF-β1 stimulation. The results showed that exogenous TGF-β1 restored the miR-206 reduced migration and invasion in ER positive BC cells. The miR-206 overexpressing cells with exogenous TGF-β1 stimulation showed increased migration and invasion compared to the miR-206 overexpressing cells without the TGF-β1 stimulation. These stimulated cells had no significant difference of migration and invasion compared to the negative control cells with normal migration and invasion properties (Figure 4). These results suggest that exogenous TGF-β1 stimulation may reverse the inhibitory effect of miR-206 overexpression.

### MiR-206 overexpression regulates phospholipase D1 (*PLD1*) gene expression in ER positive BC cells

**Figure 5:**
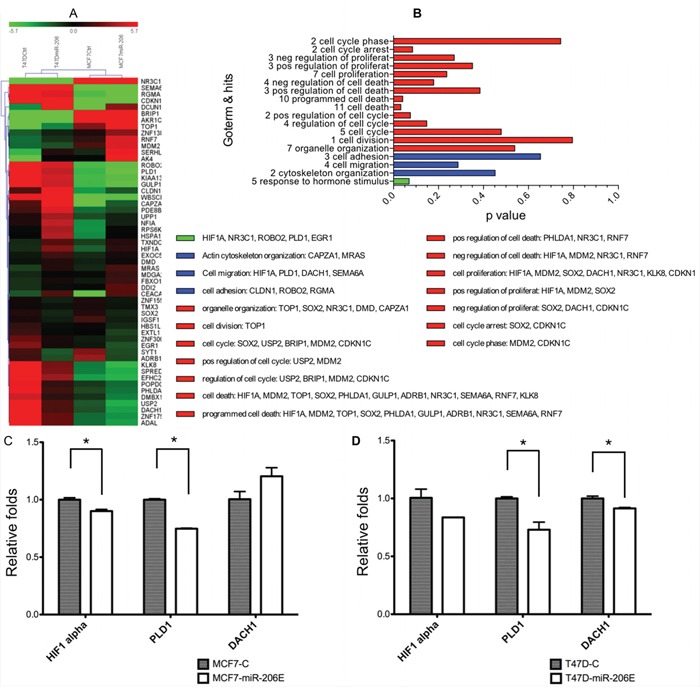
MiR-206 overexpression downregulates phospholipase D1 (PLD1) **A.** Expression heat maps of genes regulated by miR-206 overexpression, with fold change cutoff values = 1.5. **B.** Gene ontology (GO) term annotations of the genes identified by the microarray assays. **C, D.** Quantitative real-time PCR was used to validate the results of the microarray assays, and showed that PLD1 was significantly downregulated by miR-206 overexpression. The regulation by miR-206 of PLD1 was consistent and statistically significant in both MCF-7 and T47D cells. Experiments were performed in triplicate. TGF-β mRNA expression was normalized to GAPDH and is depicted as the mean ± SD.

We performed mRNA profiling to identify the migration-related downstream genes regulated by miR-206. The genes whose expression changed more than 1.5 fold in both MCF-7 and T47D miR-206 overexpressing cells were selected. In total, 55 genes were identified as downstream genes regulated by miR-206 overexpression. The gene ontology (GO) term annotation was used to classify these genes by different functions (Figure 5A). Among them, hypoxia-inducible factor (*HIF*)*1α*, *PLD1*, and Dachshund homolog 1 (*DACH1*) are classified as genes that affect cell migration (Figure 5B). We finally validated that the expression level of *PLD1* was significantly inhibited by miR-206 overexpression using qPCR in both MCF-7 and T47D cells (Figure 5C and 5D, respectively).

## DISCUSSION

The mechanism underlying the role of miR-206 in ER positive BC is still unclear, although some studies showed the miR-206 inhibitory effect on proliferation, migration, and invasion in triple negative BC [12, 13]. To the best of our knowledge, our study is the first that investigated the mechanism of miR-206 inhibitory effects in ER positive BC cells. We show that epithelial mesenchymal transition is suppressed by TGF-β signaling components.

Our data showed the inhibitory effects of miR-206 on migration, invasion, and the EMT response. MiR-206 overexpression increased the epithelial cell marker E-cadherin, in ER positive BC cells, while the mesenchymal cell markers, N-cadherin and vimentin, were decreased. As found in mesenchymal cells, the transcription factor ZEB1 was decreased significantly in both the miR-206 overexpressing cell lines, further demonstrating that the inhibitory effect of miR-206 overexpression on EMT may be mediated by suppression of the *ZEB1* gene expression. *ZEB1* suppresses the promoter activity of the E-cadherin gene *CDH1* by binding to the E-box regions of the DNA sequences that flank *CDH1* [14]. In the nucleus, *ZEB1* also can cooperate with the deacetylase sirtuin 1 to modify histone H3, and reduce binding of RNA polymerase II to the *CDH1* promoter region [15]. *ZEB1* then represses epithelial junction and polarity genes, and activates mesenchymal genes that define the EMT phenotype.

According to the predictions of TargetScan, *NRP1*, *SMAD2*, and *SMAD4* could be target genes of miR-206. They belong to the TGF-β signaling gene family, which further suggests that TGF-β signaling may mediate the EMT inhibition caused by miR-206 overexpression. Interestingly, we found both TGF-β transcription level and the TGF-β autocrine production level were downregulated in the miR-206 overexpressing cell lines. We also found that exogenous TGF-β1 could promote the migration and invasion capability, which was compromised by miR-206. These results suggest that both the autocrine production of TGF-β from cancer cells and paracrine production from the tumor microenvironment could play important roles in EMT processes. These conclusions are in agreement with the previous findings that higher autocrine TGF-β production was found in the mesenchymal cell subpopulation than in the immortalized parental human mammary epithelial cells, and this increased SMAD expression and stably maintained mesenchymal subpopulation (MSP) cells in a mesenchymal/stem cell state [16]. Previous studies showed an autocrine and paracrine TGF-β/ZEB/miR-200 signaling network that regulates the establishment and maintenance of EMT. According to these studies, the ZEB/miR-200 regulatory loop is targeted by autocrine and paracrine TGF-β [17, 18]. In contrast, our results showed that the autocrine TGF-β was targeted by miR-206 overexpression, either at the transcriptional or translational level. Although the mechanism underlying this process is still unknown, our results imply a novel regulatory link between autocrine TGF-β and miRNA. These results may provide new insight for future research on miRNAs and their related signaling pathways.

We found that *NRP1* and *SMAD2* gene expressions were decreased in miR-206 overexpressing cell lines, and further validated the direct target genes of miR-206. SMAD2 is a key signal transducer of TGF-β signaling, which generally acts by forming complexes of TGF-β receptor type II (TβRII) and TβRI. The receptor activated SMAD2 and/or SMAD3 combine with SMAD4 to form trimeric SMAD complexes that translocate to the nucleus, where they combine with DNA transcription factors at regulatory gene sequences, and induce the transcription of *ZEB1* and *Snail* genes [19, 20]. NRP1 and NRP2 are receptors for class 3 semaphorins [21, 22]. NRP1 is a co-receptor for vascular endothelial growth factor (VEGF) [23], and is an anti-angiogenic target in malignant tumors [24]. Recently, NRP1 has been shown to be a co-receptor of TGF-β, with a high affinity for TGF-β and its receptors. It combines with TβRI/II/III and forms a co-receptor complex, enhancing the affinity of TGF-β for its receptors. As a result, NRP1 might interact with other TGF-β receptors, resulting in TGF-β binding and activation, thereby facilitating cancer metastasis [25].

We confirmed that PLD1 was decreased in miR-206 overexpressing cell lines by mRNA profiling assays and qPCR. PLD1 belongs to the PLD enzyme superfamily of proteins, and contains a conserved catalytic site and a functional transphosphatidylase activity, acting at phosphodiester bonds found in a wide range of substrates. Classical PLD enzymes hydrolyze the abundant membrane lipid phosphatidylcholine to yield the second messenger phosphatidic acid (PA) and choline [26]. PLD1 has been reported to play pro-metastatic roles in multiple processes, including induced Ras and ERK activation [27], loss of polarization for epithelial cells [28], and the release of extracellular proteases that facilitate invasion [29–32]. Recently, Fite et al. identified miR-182, miR-887, miR-203, and miR-3619 as PLD1/2 targeting miRNAs. Ectopic transfection with these miRNAs could reverse PLD1/2 expression, and cause aggressiveness of post-EMT in MDA-MB-231 and BT-549 cells [33]. Our results also showed that PLD1 was regulated by miR-206; therefore, it could contribute to the inhibition of the EMT process. However, the mechanism underpinning this process is still unclear, and additional studies should investigate the exact effect of miR-206 overexpression on PLD1.

In addition to the effects of miR-206 overexpression shown in our study, other studies have also indicated that miR-206 activates apoptosis by targeting Notch3 [34], and inhibits proliferation by repressing KLF4 expression in BC cells [35]. Whether these miR-206 mediated effects are all modulated by *SMAD2*, *NRP1*, and/or *PLD1* genes is still unknown. Nevertheless, modulation of these genes may be a promising and meaningful approach to determine the mechanism of miR-206, and this process will be investigated in the future.

### Conclusion

In summary, our results demonstrate that miR-206 inhibits migration and invasion of ER positive BC cells. We report a novel inhibitory effect of miR-206 on transcription and autocrine production of TGF-β, as well as direct targeting the expression of *SMAD2* and *NRP1* in the TGF-β signaling pathway, In addition, mRNA expression assay shows that miR-206 inhibits the expression of PLD1. All of these effects suppress EMT processes (Schematic diagram 1). These results suggest that miR-206 may be an important tumor suppressor miRNA for ER positive BC. MiR-206 may therefore provide an effective therapeutic strategy for ER positive BC.

**Schematic diagram 1:**
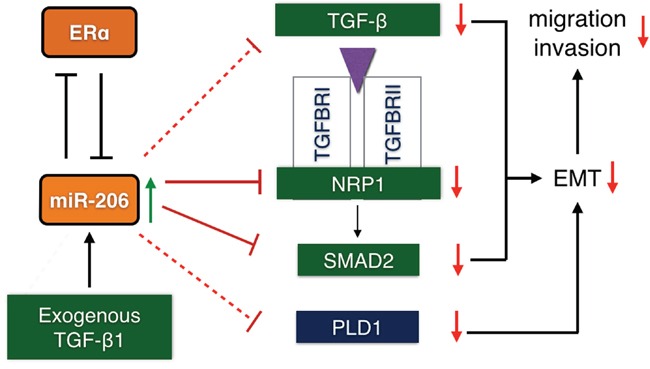
Possible regulatory mechanism of miR-206 induction of migration and invasion inhibition in ER positive BC cells through the suppression of the epithelial mesenchymal transition by targeting TGF-β signaling components MiR-206 overexpression suppresses TGF-β signaling, including the transcription and autocrine expression of TGF-β, and the expression of the *NRP1* and *SMAD2* genes. Phospholipase D1 (PLD1) expression is also suppressed. This results in the inhibition of the EMT response, thereby inhibiting the migration and invasion of ER positive BC cells. (Solid line: 3′-UTR targeted suppression; dotted line: suppression by an unknown mechanism.

## MATERIALS AND METHODS

### Cell culture

The human ER positive breast cancer cell lines MCF-7 and T47D were obtained from the American Type Culture Collection (Manassas, VA, USA). All cells were maintained at 37°C in an atmosphere of 5% CO_2_, and were cultured in Dulbecco's modified Eagle's medium (DMEM; Hyclone, Logan, UT, USA) supplemented with 10% fetal bovine serum (FBS) (Gibco-Thermo Fisher Scientific, Waltham, MA, USA). All BC (MCF7-miR-206E and T47D-miR-206E) cell lines stably expressing miR-206, and the respective negative control cells were cultured in DMEM supplemented with 10% FBS (Gibco-Thermo Fisher Scientific) and 0.5 μg/mL puromycin.

### Construction of miR-206 stably overexpressing cells

The 293T cells were co-transfected with PMD2G, psPAX2, and pLV-miRNA-206 expression vectors or the pLV-miRNA-control expression vector (Biosettia, San Diego, CA, USA) using Lipofectamine^®^ 2000 (Invitrogen Life Sciences, Carlsbad, CA, USA). After 48 hours, culture supernatants were collected, passed through 0.45 μm filters, and mixed with fresh media (1:1) and polybrene (8 μm/mL) to infect MCF-7 and T47D cells. Cells with overexpression of miR-206 were designated as MCF7-miR-206E and T47D-miR-206E, and the control cells infected with pLV-miRNA-contral expression vector were designated as MCF7-C and T47D-C. Stable cell lines were established using puromycin selection.

### RNA extraction and qPCR

Total RNA was extracted using TRIzol^®^ reagent (Invitrogen) and miRNeasy kits (Qiagen, Hilden, Germany) according to the manufacturer's protocol. The miRNA was converted to cDNA by using the PrimeScript^®^ RT reagent kit (TaKaRa, Tokyo, Japan) according to the manufacturer's protocol. MiR-206, TGF-β mRNAs, and genes of the TGF-β signaling pathway family were analyzed by qPCR in triplicate using 100 ng cDNA and SYBR^®^ Premix Ex Taq (TaKaRa) on an Applied Biosystems 7500 Fast Real-Time PCR System (Applied Biosystems, Foster City, CA, USA). The miRNA and mRNA fold changes were determined using the ΔΔCT method with normalization to U6 snRNA and GAPDH, respectively. The primers are listed in Supplementary Methods (Table S1).

### *In vitro* Transwell^®^ migration and invasion assays

Cells (1.0 × 10^5^ cells) were plated into the top of the Transwell^®^ chamber (24-well insert; pore size, 8.0 μm; BD Biosciences, San Jose, CA, USA) for migration assays, and the same number of cells was plated into the top chamber over a Matrigel^®^-coated filter (24-well insert; pore size, 8.0 μm; BD Biosciences) for invasion assays. Both top chambers were filled with 0.5 mL of serum free DMEM medium, and 1 mL of DMEM supplemented with 10% FBS was used as the chemoattractant in the lower chambers. After 24 hours, for the MCF-7 cells and 36 hours for the T47D cells, the cells that did not migrate or invade through the membranes were removed, and cells on the underside of the membrane were fixed in anhydrous methanol, and then stained with 0.1% crystal violet. The chambers with stained cells were washed with 1× phosphate-buffered saline (PBS) and counted.

### Enzyme-linked immunosorbent assay (ELISA)

Cells (1.0 × 10^5^) overexpressing miR-206 and negative control cells were plated in 24-well plates. The culture supernatants were collected and autocrine TGF-β levels were measured using the Quantikine^®^ Human TGF-β1 immunoassay (R&D Systems, Minneapolis, MN, USA) using the manufacturer's protocol. Each experiment was performed in three wells and was repeated three times.

### mRNA microarray analyses

The Affymetrix PrimeView™ human gene expression microarray (Affymetrix, Santa Clara, CA, USA) was used in this study. Total RNA was extracted from four independent cultures of MCF7-miR-206E cells, MCF7-C cells, T47D-miR-206E cells, and T47D-C cells. Microarray hybridization, data acquisition, and analyses were performed at Shanghai Biotechnology Corporation (Shanghai, China). Statistically significant differential expression was determined according to the 1.5× fold changes in transcript levels between both the MCF-7 group and the T47D groups. According to the functional GO annotation of the genes, the microarray analyses indicated that miR-206 overexpression upregulated and downregulated downstream genes.

### Exogenous TGF-β1 stimulation

One day before stimulation, 3 × 10^5^ cells were plated into six-well culture plates to achieve 70%–80% confluency. Cells were stimulated with varying concentrations of recombinant human TGF-β1 (HumaXpress™; Humanzyme, Chicago, IL, USA). The culture medium was replaced with fresh medium after 48 hours, and the recombinant human TGF-β1 was added to each well with the same concentration added at each time period. After 48 hours, the cells were collected for subsequent experiments.

### Western blot assays

Cells (3 × 10^5^) were plated into six-well culture plates to achieve 70%–80% confluency, and were washed in PBS and suspended in 100 μL of RIPA buffer (Pierce, Dallas, TX, USA). Supernatant protein concentrations were determined using the BCA protein assay kit (Pierce). Supernatant samples containing 30 μg total protein were resolved by 10% or 12.5% sodium dodecyl sulfate polyacrylamide gel electrophoresis (SDS-PAGE) depending on the molecular weights of the target proteins, and were transferred to immobilon-P polyvinylidene difluoride (PVDF) membranes (Millipore, Billerica, MA, USA) by electroblotting, and then probed with anti-E-cadherin (Cat# sc-21791, Santa Cruz Biotechnology, Santa Cruz, CA, USA), anti-N-cadherin (Cat# sc-53488, Santa Cruz Biotechnology), anti-Vimentin (Cat# gtx100619, GeneTex, Irvine, CA, USA), anti-SNAIL1 (Cat# gtx125918, GeneTex), anti-Twist (Cat# gtx127310, GeneTex), anti-ZEB1 (Cat# gtx105278, GeneTex), anti-STC2 (Cat# sc-14352, Santa Cruz Biotechnology), anti-ANXA2 (Cat# sc-9061, Santa Cruz Biotechnology), anti-NRP1 (Cat# 3725, Cell Signaling Technology, Danvers, MA, USA), anti-SMAD2 (Cat#: 3103, Cell Signaling Technology), or anti-GAPDH (Cell Signaling Technology) antibodies. Membranes were incubated with horseradish peroxidase-conjugated secondary antibodies. Blots were developed using an ECL kit (Merck Millipore, Billerica, MA, USA).

### NRP1 and SMAD2 3′-UTR dual luciferase reporter assays

To generate the dual luciferase reporter vectors, wild type or mutant human *NRP1* 3′-UTRs and wild type or mutant human SMAD2 3′-UTR segments (See Supplementary Table 1 for sequences) were amplified by PCR from cDNAs and cloned into the luciferase reporter vector GV272 (Genechem, Shanghai, China). The 293T cells were plated into 24-well culture plates to achieve 40%–50% confluency. The miR-206 mimic (40 nM) or NC-mimic (GenePharma, Shanghai, China) and 200 ng 3′-UTR luciferase construct were co-transfected using Lipofectamine^®^ 2000 (Invitrogen), according to the manufacturer's protocol. Cells were cultured for 24 hours after transfection and the luciferase activity was measured using the Dual-Luciferase reporter assay system (Promega, Sunnyvale, CA, USA). Firefly luciferase activity was normalized to *Renilla* luciferase activity. Experiments were performed in triplicate and repeated in triplicate.

### Statistical analyses

Statistical analyses were performed using SPSS version 16.0. software (SPSS, Chicago, IL, USA). Differences among variables were analyzed by the two-tailed Student's *t*-test. Data were presented as the mean ± SD unless otherwise indicated. Statistical significance was defined as *P* < 0.05.

## SUPPLEMENTARY DATA FIGURE AND TABLE



## References

[1] Siegel R, Ma J, Zou Z, Jemal A (2014). Cancer statistics, 2014. CA.

[2] Cancer incidence in five continents (2008). Volume IX. IARC Sci Publ.

[3] Preston-Martin S, Pike MC, Ross RK, Jones PA, Henderson BE (1990). Increased cell division as a cause of human cancer. Cancer research.

[4] Lu J, Getz G, Miska EA, Alvarez-Saavedra E, Lamb J, Peck D, Sweet-Cordero A, Ebert BL, Mak RH, Ferrando AA, Downing JR, Jacks T, Horvitz HR, Golub TR (2005). MicroRNA expression profiles classify human cancers. Nature.

[5] Iorio MV, Ferracin M, Liu CG, Veronese A, Spizzo R, Sabbioni S, Magri E, Pedriali M, Fabbri M, Campiglio M, Menard S, Palazzo JP, Rosenberg A, Musiani P, Volinia S, Nenci I (2005). MicroRNA gene expression deregulation in human breast cancer. Cancer research.

[6] Sempere LF, Christensen M, Silahtaroglu A, Bak M, Heath CV, Schwartz G, Wells W, Kauppinen S, Cole CN (2007). Altered MicroRNA expression confined to specific epithelial cell subpopulations in breast cancer. Cancer research.

[7] Blenkiron C, Goldstein LD, Thorne NP, Spiteri I, Chin SF, Dunning MJ, Barbosa-Morais NL, Teschendorff AE, Green AR, Ellis IO, Tavare S, Caldas C, Miska EA (2007). MicroRNA expression profiling of human breast cancer identifies new markers of tumor subtype. Genome biology.

[8] Tavazoie SF, Alarcon C, Oskarsson T, Padua D, Wang Q, Bos PD, Gerald WL, Massague J (2008). Endogenous human microRNAs that suppress breast cancer metastasis. Nature.

[9] Adams BD, Furneaux H, White BA (2007). The micro-ribonucleic acid (miRNA) miR-206 targets the human estrogen receptor-alpha (ERalpha) and represses ERalpha messenger RNA and protein expression in breast cancer cell lines. Molecular endocrinology (Baltimore, Md).

[10] Kondo N, Toyama T, Sugiura H, Fujii Y, Yamashita H (2008). miR-206 Expression is down-regulated in estrogen receptor alpha-positive human breast cancer. Cancer research.

[11] Yoshimoto N, Toyama T, Takahashi S, Sugiura H, Endo Y, Iwasa M, Fujii Y, Yamashita H (2011). Distinct expressions of microRNAs that directly target estrogen receptor alpha in human breast cancer. Breast cancer research and treatment.

[12] Rebbeck TR, Lynch HT, Neuhausen SL, Narod SA, Van't Veer L, Garber JE, Evans G, Isaacs C, Daly MB, Matloff E, Olopade OI, Weber BL (2002). Prophylactic oophorectomy in carriers of BRCA1 or BRCA2 mutations. The New England journal of medicine.

[13] Clemons M, Goss P (2001). Estrogen and the risk of breast cancer. The New England journal of medicine.

[14] Remacle JE, Kraft H, Lerchner W, Wuytens G, Collart C, Verschueren K, Smith JC, Huylebroeck D (1999). New mode of DNA binding of multi-zinc finger transcription factors: deltaEF1 family members bind with two hands to two target sites. The EMBO journal.

[15] Romano LA, Runyan RB (2000). Slug is an essential target of TGFbeta2 signaling in the developing chicken heart. Developmental biology.

[16] Scheel C, Eaton EN, Li SH, Chaffer CL, Reinhardt F, Kah KJ, Bell G, Guo W, Rubin J, Richardson AL, Weinberg RA (2011). Paracrine and autocrine signals induce and maintain mesenchymal and stem cell states in the breast. Cell.

[17] Gregory PA, Bracken CP, Smith E, Bert AG, Wright JA, Roslan S, Morris M, Wyatt L, Farshid G, Lim YY, Lindeman GJ, Shannon MF, Drew PA, Khew-Goodall Y, Goodall GJ (2011). An autocrine TGF-beta/ZEB/miR-200 signaling network regulates establishment and maintenance of epithelial-mesenchymal transition. Molecular biology of the cell.

[18] Xu Q, Wang L, Li H, Han Q, Li J, Qu X, Huang S, Zhao RC (2012). Mesenchymal stem cells play a potential role in regulating the establishment and maintenance of epithelial-mesenchymal transition in MCF7 human breast cancer cells by paracrine and induced autocrine TGF-beta. International journal of oncology.

[19] Kang Y (2006). Pro-metastasis function of TGFbeta mediated by the Smad pathway. Journal of cellular biochemistry.

[20] Peinado H, Quintanilla M, Cano A (2003). Transforming growth factor beta-1 induces snail transcription factor in epithelial cell lines: mechanisms for epithelial mesenchymal transitions. The Journal of biological chemistry.

[21] Kolodkin AL, Levengood DV, Rowe EG, Tai YT, Giger RJ, Ginty DD (1997). Neuropilin is a semaphorin III receptor. Cell.

[22] Chen H, Chedotal A, He Z, Goodman CS, Tessier-Lavigne M (1997). Neuropilin-2, a novel member of the neuropilin family, is a high affinity receptor for the semaphorins Sema E and Sema IV but not Sema III. Neuron.

[23] Soker S, Takashima S, Miao HQ, Neufeld G, Klagsbrun M (1998). Neuropilin-1 is expressed by endothelial and tumor cells as an isoform-specific receptor for vascular endothelial growth factor. Cell.

[24] Jubb AM, Strickland LA, Liu SD, Mak J, Schmidt M, Koeppen H (2012). Neuropilin-1 expression in cancer and development. The Journal of pathology.

[25] Glinka Y, Stoilova S, Mohammed N, Prud'homme GJ (2011). Neuropilin-1 exerts co-receptor function for TGF-beta-1 on the membrane of cancer cells and enhances responses to both latent and active TGF-beta. Carcinogenesis.

[26] Jenkins GM, Frohman MA (2005). Phospholipase D: a lipid centric review. Cellular and molecular life sciences: CMLS.

[27] Sulzmaier FJ, Valmiki MK, Nelson DA, Caliva MJ, Geerts D, Matter ML, White EP, Ramos JW (2012). PEA-15 potentiates H-Ras-mediated epithelial cell transformation through phospholipase D. Oncogene.

[28] Gloerich M, ten Klooster JP, Vliem MJ, Koorman T, Zwartkruis FJ, Clevers H, Bos JL (2012). Rap2A links intestinal cell polarity to brush border formation. Nature cell biology.

[29] Kang DW, Hwang WC, Park MH, Ko GH, Ha WS, Kim KS, Lee YC, Choi KY, Min DS (2013). Rebamipide abolishes Helicobacter pylori CagA-induced phospholipase D1 expression via inhibition of NFkappaB and suppresses invasion of gastric cancer cells. Oncogene.

[30] Kang DW, Park MH, Lee YJ, Kim HS, Lindsley CW, Alex Brown H, Min do S (2011). Autoregulation of phospholipase D activity is coupled to selective induction of phospholipase D1 expression to promote invasion of breast cancer cells. International journal of cancer.

[31] Kato Y, Lambert CA, Colige AC, Mineur P, Noel A, Frankenne F, Foidart JM, Baba M, Hata R, Miyazaki K, Tsukuda M (2005). Acidic extracellular pH induces matrix metalloproteinase-9 expression in mouse metastatic melanoma cells through the phospholipase D-mitogen-activated protein kinase signaling. The Journal of biological chemistry.

[32] Reich R, Blumenthal M, Liscovitch M (1995). Role of phospholipase D in laminin-induced production of gelatinase A (MMP-2) in metastatic cells. Clinical & experimental metastasis.

[33] Fite K, Gomez-Cambronero J (2016). Downregulation of miRs 203, 887, 3619 and 182 prevent vimentin-triggered, phospholipase D (PLD)-mediated cancer cell invasion. The J Biol Chem.

[34] Song G, Zhang Y, Wang L (2009). MicroRNA-206 targets notch3, activates apoptosis, and inhibits tumor cell migration and focus formation. The Journal of biological chemistry.

[35] Lin CC, Liu LZ, Addison JB, Wonderlin WF, Ivanov AV, Ruppert JM (2011). A KLF4-miRNA-206 autoregulatory feedback loop can promote or inhibit protein translation depending upon cell context. Molecular and cellular biology.

