# Supplementary material for: MiR-206 suppresses epithelial mesenchymal transition by targeting TGF-β signaling in estrogen receptor positive breast cancer cells
Source: Oncotarget. 2016 Mar 21;7(17):24537–48. doi: 10.18632/oncotarget.8233 (PMC5029720; doi:10.18632/oncotarget.8233)
Supplement: Supplementary file 1 [file oncotarget-07-24537-s001.pdf]

SUPPLEMENTARY DATA

Primers for RT-PCR and qPCR

miR-206: sense primer: ACACTCCAGCTGGGTGGAATGTAAGGAAGT;

U6:sense primer: ACACTCCAGCTGGGCGCAAATTCGTGAAGC;

Universal anti-sense primer for microRNA: CTCAACTGGTGTCGTGGAGTCGG;

TGF-β: sense primer: GGCGATACCTCAGCAACCG;

anti-sense primer: AAGGCGAAAGCCCTCAAT;

HIF1α: sense primer: TACTCAAAGTCGGACAGCC;

anti-sense primer: CAAAGCGACAGATAACACG;

PLD1: sense primer: GTCATCCACCGTCTATTTG;

anti-sense primer: CTGTGCTCATTGTCGTCCC;

DACH1: sense primer: ATCCAGCCAGGAGTGAACC;

anti-sense primer: GAATGCGGCATGATGTGAG

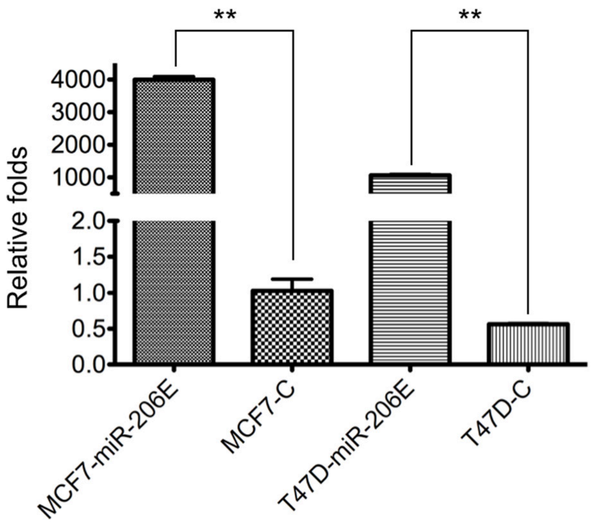

Supplementary Figure S1: Stable transfection of miR-206 significantly promoted miR-206 expression level in both MCF-7 and T47D cells. (**: *P*< 0.001). Experiments were performed 3 times. TGF-β mRNA expression was normalized to U6 and depicted as the mean ± S.D.

Supplementary Table S1: Wildtype and mutant 3'-UTR segments for NRP1 and SMAD2

NRP1-3'UTR-WILDTYPE	AAATTGTTGTTTCAAAATGAACAGCGAAGCCTTAACT<br>TTAAATGACATTTGTATTCTCAGACACTGAGTAGCATAAAAACCACATAGAA<br>CTGAACTGTAACTTAAATTCCAAACTATGACTACTACATTCCAAAGAAACA<br>GTTGAATTAAACATTTTCATAAAATATCCCACAAA
NRP1-3'UTR-MUTANT	AAATTGTTGTTTCAAAATGAACAGCGAAGCCTTA<br>ACTTTAAATGACATTTGTATTCTCAGACACTGAGTAGCATAAAAACCA<br>CATAGAACTGAACTGTAACTTAAATTCCAAACTATGACTACTCACAA<br>AACAAGAAACAGTTGAATTAAACATTTTCATAAAATATCCCACAAA
SMAD2-3'UTR-WILDTYPE	TGAGGGTGGCGTAGGTTTATGTTGGGATATA<br>AAGTAACACAAGACAATCTTCACTGTACATAAAATATGTCTTCATG<br>TACAGTCTTTACTTTAAAAGCTGAACATTCCAATTTGCGCCTTCCC<br>TCCCAAGCCCCTGCCCACCAAGTATCTCTTTAGATATCTAGTCTGT<br>GGACATGAACAATGAATACTTTTTTCTTACTCTGATCGAA
SMAD2-3'UTR-MUTANT	TGAGGGTGGCGTAGGTTTATGTTGGGATATA<br>AAGTAACACAAGACAATCTTCACTGTACATAAAATATGTCTTCATG<br>TACAGTCTTTACTTTAAAAGCTGACACAAAACATTTGCGCCTTCC<br>CTCCCAAGCCCCTGCCCACCAAGTATCTCTTTAGATATCTAGTCT<br>GTGGACATGAACAATGAATACTTTTTTCTTACTCTGATCGAA
